# Two decades of studying non-covalent biomolecular assemblies by means of electrospray ionization mass spectrometry

**DOI:** 10.1098/rsif.2011.0823

**Published:** 2012-02-08

**Authors:** Gillian R. Hilton, Justin L. P. Benesch

**Affiliations:** Department of Chemistry, Physical and Theoretical Chemistry Laboratory, University of Oxford, South Parks Road, Oxford OX3 1QZ, UK

**Keywords:** mass spectrometry, ion mobility, protein assembly, non-covalent complex, hybrid structural biology

## Abstract

Mass spectrometry (MS) is a recognized approach for characterizing proteins and the complexes they assemble into. This application of a long-established physico-chemical tool to the frontiers of structural biology has stemmed from experiments performed in the early 1990s. While initial studies focused on the elucidation of stoichiometry by means of simple mass determination, developments in MS technology and methodology now allow researchers to address questions of shape, inter-subunit connectivity and protein dynamics. Here, we chart the remarkable rise of MS and its application to biomolecular complexes over the last two decades.

## 1. Introduction

Since its invention at the beginning of the last century, mass spectrometry (MS) has been considered an essential tool for chemists and physicists alike, primarily being used to analyse small molecules and volatile compounds. At the end of the 1980s, its utility broadened dramatically, as its application to problems in biology began in earnest. The first reports on the MS of non-covalent complexes appeared in the literature in the early 1990s, with the initial studies focusing on protein–ligand complexes quickly joined by those in which protein–protein interactions were maintained [1]. Since those early days considerable improvements in instrument technology and experimental methodology have dramatically increased the range of protein assemblies amenable to MS analysis [2]. As a result, 20 years after the initial reports, assemblies as large as intact viruses; of as many components as ribosomes; as hydrophobic as membrane protein complexes; as heterogeneous as amyloidogenic oligomers; and as dynamic as molecular chaperones have all been successfully interrogated [3–7]. MS has therefore evolved to impact a wide range of applications in structural biology.

**Figure 1.**
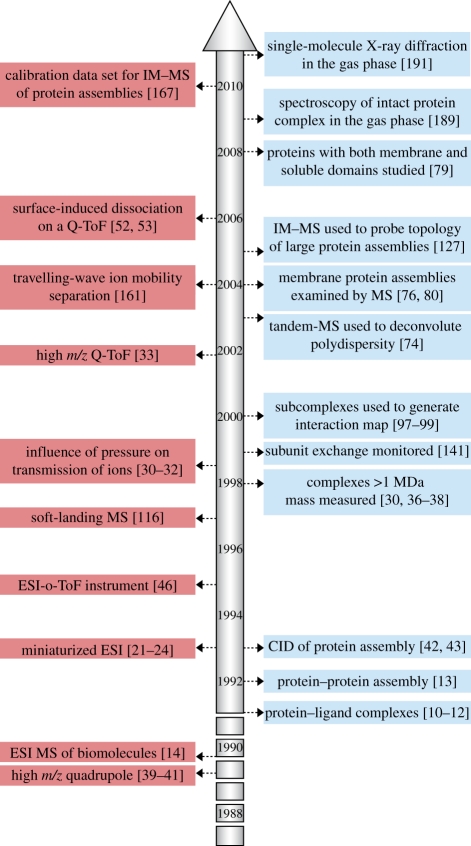
Some important milestones in the developments in MS instrumentation for the study of macromolecular assemblies (left-hand side), and the subsequent methodologies they enabled (right-hand side). The first non-covalent complexes were measured by means of MS in 1991, and the following two decades have seen dramatic progress in both the technology and its application to problems in structural biology.

In this historical perspective, we chart some of the milestones in MS development as they pertain to the study of non-covalent complexes, and the novel applications they have enabled (figure 1). While multiple MS approaches can inform on such assemblies [4], we focus here on those in which they are examined intact in the gas phase. We also describe the current state of the art in MS instrumentation and sample preparation, and direct to the relevant literature. Having described the past and present of the MS of non-covalent complexes, we allow ourselves to indulge in speculation as to what the future of the field might hold. As such, our intention is that this review serves as a ‘primer’ for scientists new to the field, providing an entry point to the literature. This is inevitably a subjective undertaking, and though we have endeavoured to be as comprehensive as possible, we apologize in advance for any omissions and hope the interested reader will soon fill in the gaps.

## 2. The development of MS for structural biology

### 2.1. The initial discoveries

In the early 1990s, a series of seminal studies demonstrated that bimolecular complexes held together by non-covalent interactions could be transferred into the vacuum of the mass spectrometer and analysed. While the preservation of non-covalent interactions, in the form of salt and solvent bound to proteins, had been observed a few years previously, this detection of specific and biologically relevant complexes represented a major breakthrough [1,8,9].

The earliest of these reports appeared in the literature in 1991, concerning the receptor–ligand binding of FK binding protein and macrolides [10]; the enzyme–substrate pairing of lysozyme and a hexasaccharide [11]; and the haem-binding of myoglobin [12]. These studies were soon followed by an array of other examples in which non-covalent interactions were maintained in the gas phase including the notable first measurement of a protein–protein assembly, the human immunodeficiency virus protease dimer [13]. These, and other, pioneering studies are described in a comprehensive review [1] and, together with the realization that proteins perform their cellular roles not in isolation but rather in complex with a multitude of other biomolecules, paved the way for MS in structural biology [8,9].

### 2.2. Optimizing transfer into the gas phase

While these reports clearly showed the promise of MS for studying protein assemblies, much work remained in the development of both technology and methodology. Electrospray ionization (ESI) was an essential development in MS as it allowed the transfer of protein from solution molecules into gas-phase ions. The above studies all employed ESI, which in the late 1980s had been shown to enable the MS analysis of intact protein chains [14]. Such ‘soft’ or ‘gentle’ ionization was in stark contrast to previous approaches which caused extensive covalent bond fragmentation, and were effectively limited to molecular weights on the order of 10 kDa [15].

#### 2.2.1. Electrospray mechanism

ESI is achieved by applying a potential difference between the inlet of the mass spectrometer and a conductive capillary containing the analyte solution [16]. This results in the production of charged droplets at the end of the capillary which evaporate solvent as they pass into the vacuum of the mass spectrometer. The droplets shrink until they reach the Rayleigh limit, the point at which the surface tension holding them together equals the Coulombic repulsion between the charges on their surface, and droplet fission occurs. Successive rounds of evaporation and fission occur until an analyte ion is formed via one of two different mechanisms. Analyte ions formed by the ‘ion evaporation model’ are expelled directly from the droplets [17], whereas those resulting from the ‘charged-residue model’ arise as the end product after droplet fisson and solvent evaporation processes have reached exhaustion [18]. The current evidence suggests that folded protein ions are generated according to the latter [19].

#### 2.2.2. Nanoelectrospray and native MS

**Figure 2.**
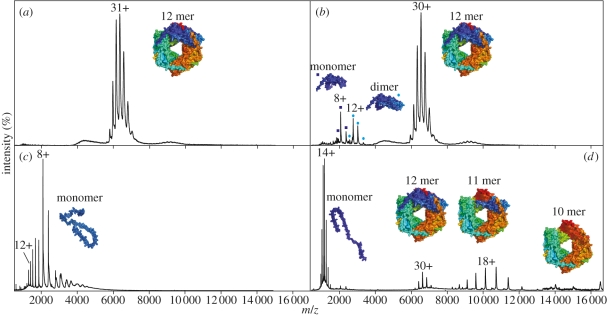
Four example mass spectra of the same protein complex subjected to different experimental conditions. These spectra show the complex intact; solution adjusted conditions to reveal the presence of monomers and dimers; denaturing solution conditions (addition of organic solvent and acid); and finally MS activating conditions showing the gas phase fragmentation. The protein complex, a small Heat Shock Protein (sHSP) *Ta*HSP16.9, is an oligomeric species comprising six dimeric building blocks to form a 12 mer (see inset 2(*a*)). (*a*) Mass spectrum of *Ta*HSP16.9 under near ‘native’ conditions applying mild instrument conditions such as low collision voltages and ion guide pressures optimized to allow the transmission of the ions through the mass spectrometer. The spectrum shows a narrow charge series (30+ to 34+) corresponding to 202 237 Da, the mass of the intact 12 mer of HSP16.9 (see the inset). The multiple charge states are a direct result of the distribution of charges on the nESI droplet. (*b*) Example spectrum showing the effects of solution phase manipulation by the addition of isopropanol 10% (v/v). The observation of dimer in the spectrum suggests a destabilization of the dimer–dimer interfaces, the interactions required to construct the intact 12 mer. (*c*) Denaturing conditions (50% acetonitrile and 0.1% formic acid (v/v), aqueous) reduces the 12 mer to monomeric units with a broad charge state distribution. The larger the surface area exposed, the more charges can be accommodated, and therefore a narrow distribution of low average charge suggests a folded protein state whereas an unfolded/disordered protein will have an extended highly charged distribution. (*d*) Spectrum under activating conditions in which monomers are ejected from the intact 12 mer to form an 11 mer and subsequently a 10 mer. Expelled monomers can be observed at low *m/z*. Unless otherwise stated, all spectra were obtained on a modified Q-ToF instrument (Waters, Manchester), as described previously [27], with a 10 µM monomer concentration of *Ta*HSP16.9 in 200 mM ammonium acetate pH 6.9.

The use of ESI was not without its challenges for the analysis of biomolecules. Experiments required substantial sample volumes, and typically relied on a combination of organic solvents, acids and high temperatures to aid the desolvation and droplet fission processes, and thereby allow reliable ion production [20]. These conditions are generally not compatible with the preservation of biomolecular complexes in solution, and therefore limit the scope of conventional ESI in structural biology [2]. To overcome these difficulties miniaturized ESI [21–23] sources were designed which, by virtue of a smaller capillary diameter, lower the flow rate to nl min^−1^ levels and therefore reduce sample consumption to only a few µl. The reduced flow rate has the added benefit of producing smaller initial droplet sizes [24], which both increases sensitivity and salt tolerance [25], and crucially negates the need for organic co-solvents and high interface temperatures. In this way, the examination of proteins in neutral aqueous buffers in which their structure is preserved has become possible, in a strategy often termed ‘Native MS’ [9,26] (figure 2*a*).

### 2.3. Transmitting and analysing large ions

The application of nano-ESI (nESI) not only enabled proteins to be analysed in their native form but also brought new challenges to MS technology. In order that the large biomolecular complexes which could now be ionized (up to ≈100 MDa [28]) might be separated and measured by the mass spectrometer, significant technological improvements were required.

Experiments have shown that increasing the pressure in the early vacuum stages of the instrument dramatically improved the transmission of large ions [29–32]. This is owing to the increased number of collisions experienced by the analyte ions acting to focus them onto the appropriate trajectory in the instrument [33]. By dampening the trajectories of the ions, the literature of which has been discussed in detail [2,34], a dramatic reduction in the loss of high mass ions is observed. This discovery brought with it new and exciting opportunities, and before the turn of the century, species over 1 MDa were being successfully transmitted through the mass spectrometer [29,35–37].

#### 2.3.1. Separation at high *m*/*z*

Many of the early studies of non-covalent complexes used triple-quadrupole mass spectrometers. These instruments have the advantage of allowing tandem-MS experiments (§2.4) but are typically limited to a maximum acquisition range of approximately 4000 *m/z*. To overcome this limitation, mass spectrometers were built incorporating a quadrupole operating at a lower radio-frequency, thereby allowing the separation of higher *m/z* species [38–40]. While this enabled the analysis of proteins in the 100 kDa range [41,42], the low resolving power at high *m/z* represented a considerable disadvantage.

In contrast, time-of-flight (ToF) mass analysers have a theoretically unlimited mass range and, when operated with a reflectron [43], can achieve high mass resolution and sensitivity on a very fast timescale. To take full advantage of the capabilities of ESI, novel instrumentation geometry was designed in which the continuous beam of ions was pulsed orthogonally into the ToF, allowing the identification of peaks well above 5000 *m/z* [44–46]. An early example demonstrating the utility of combining nESI with ToF was a study of the enzyme 4OT, where the sensitivity and resolution afforded by MS settled a conflict in the field as to the protein's oligomeric state [47]. This heralded an important shift in the MS of protein assemblies from being a method which was considered a technical curiosity to one which could be used to provide novel structural biology insight.

While other mass analysers have been used to examine intact protein complexes [48], the ‘hybrid’ Q-ToF has been the favoured instrument geometry for about a decade [32], capitalizing on the *m/z*-range benefits of ToF with the selection abilities of a quadrupole (Q) [49]. The decreased resolution of this first analyser does not impact on the final spectrum, as this is determined by the subsequent ToF stage. The great advantage of this instrument configuration is the ability to perform tandem-MS on high mass species to help elucidate their composition [32,34,50].

### 2.4. Gas phase manipulations

With the technology allowing the transmission of intact protein assemblies through the mass spectrometer and their mass measurement with unparalleled accuracy, attention shifted towards devising means for their gas-phase disassembly, such that their constituents might be probed. Multiple activation approaches have been developed to achieve this, including by impacting a surface [51,52], interactions with electrons [53,54] and absorption of infrared photons [55].

All of these approaches have their advantages, but collision-induced dissociation (CID) remains the most commonly applied approach for activation. CID was developed in the 1960s and is based on the analyte ions colliding with inert gas, resulting in activation as their translational energy is converted into internal modes by many consecutive collision events [56].

#### 2.4.1. Effects of collisional activation

CID is typically performed in two regions of the mass spectrometer where the pressure is relatively high: in the source region, or in a specifically designed collision cell. Irrespective of the location within the instrument, this thermal heating incurs the same consequences on biomolecular assemblies: cleaning, restructuring, unfolding, dissociating and fragmenting [57].

Under non-denaturing conditions the measured mass of a large protein complex is higher than that calculated from the sequence alone, owing to the adduction of salt and solvent during the ESI process [58]. The process of activation results in ‘cleaning’ of the protein by removal of these bound species, and thereby provides an increase in effective mass accuracy and resolution in the spectra [59]. Further increases in internal energy can lead to structural distortions of the protein assembly, such as the collapse of cavities within the structure [60].

At elevated activation conditions the individual protein chains begin to unfold, a process which continues until a threshold is reached and a subunit is ejected from the complex [61]. Dissociation occurring via unfolding leads to the expelled subunit typically carrying a proportion of the charge disproportionately high relative to its mass [41,62–64]. This process can occur repeatedly, with multiple subunits being removed sequentially from the assembly [65]. At the highest energies the unfolded monomers can undergo covalent fragmentation after their expulsion from the complex [66]. There has been considerable interest in recent years to manipulate the pathway of dissociation in order to obtain more information, including the adjustment of charge states [67,68], or effecting ion activation through collision with a surface [52,69]. The latter approach, surface-induced dissociation, shows particular promise for the analysis of protein assemblies in potentially allowing the determination of the building blocks of the oligomers [70].

#### 2.4.2. Deconvoluting heterogeneity with tandem-MS

The ability of gas-phase activation to afford information on the components within a protein assembly is particularly powerful when employed in the form of tandem-MS (also referred to as MS/MS). In this approach ions can be selectively subjected to CID, and the resulting dissociation products measured in a second analysis stage [71–73]. This approach became established in the study of protein assemblies after the development of Q-ToF instruments with a high-*m/z* quadrupole [32,34,50].

**Figure 3.**
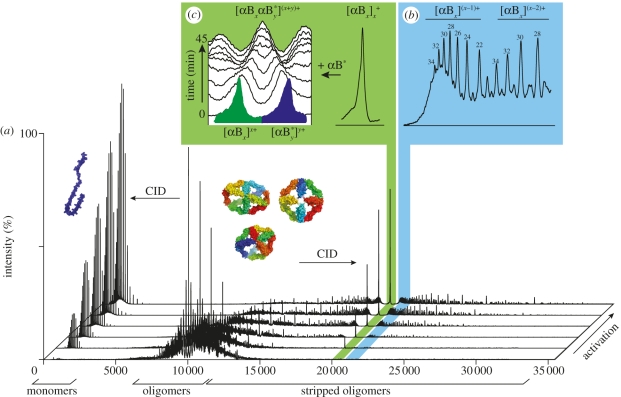
Nano-ESI MS of the heterogeneous ensemble populated by αB-crystallin. (*a*) Spectra obtained under conditions of increasing activation, indicated on the *z*-axis, in which ions are subjected to energetic collisions with argon atoms. The peaks at low *m/z* are from monomers and those at higher *m/z* are their complementary stripped oligomers. The peaks in the region between 18 000 and 24 000 *m/z* correspond to oligomers stripped of two monomers which have sufficient resolution to allow the identification and relative quantification of their individual species (*b*). The values for ‘*x*’ are indicated above each ‘even’ peak with the black dot showing the corresponding ‘odd’ stoichiometries. The peak highlighted in green at *m/z* ∼20 200 corresponds to all αB-crystallin doubly stripped oligomers carrying the equivalent number of charges as subunits (*c*). When a sample of αB-crystallin is mixed and incubated with its isotopically labelled equivalent (blue), the gradual disappearance of the homo-oligomers and the concomitant formation of the hetero-oligomer allows the quaternary dynamics and architecture to be obtained. All spectra were obtained on a modified Q-ToF instrument (Waters, Manchester), as described previously [74].

The high resolution of MS can be exploited to allow the different components within a mixture to be individually interrogated. Furthermore, in cases where the MS spectrum cannot be unambiguously assigned, knowledge that dissociation products must be complementary leads to tandem-MS aiding the assignment [32]. Another advantage of the nature of gas-phase dissociation is that the removal of highly charged monomers results in an effective charge reduction of the parent oligomers [65]. This has been exploited to quantify the relative distribution of the species comprising polydisperse ensembles which cannot be deconvoluted by MS alone [74] (figure 3).

#### 2.4.3. Examining membrane proteins

Membrane-associated proteins are among the most challenging of protein systems for structural biology owing to their solubility requirements. While a vacuum can be regarded as hydrophobic and therefore a suitable environment in which to study such proteins [75], transferring them intact into the mass spectrometer has been a challenge. The first approach which brought success was to prepare a protein in a concentration of a detergent sufficient to solubilize the exposed hydrophobic surfaces, but not so high as to obscure the signal corresponding to protein [76].

The observation that detergent micelles could apparently be maintained in the gas phase [77,78] led to the development of an alternative strategy. In this approach, the protein assembly is encapsulated within a micelle to enable its transfer into the gas phase, whereupon the detergents are subsequently removed by collisional activation [79,80]. This process has the potential to be applicable to various membrane protein systems [81], and recent evidence suggests that it might even be possible to remove the detergent without excessive structural rearrangement of the protein [82]. Considering the importance of membrane proteins as drug targets, perhaps the most exciting aspect of this application is the ability to detect the presence of small molecules, and their influence on the structure and stability of the complex [83].

### 2.5. Solution phase manipulations

In the early 1990s, it was noted that solution conditions could affect the ESI mass spectra of proteins. Reduction of disulphide bonds [84], manipulation of pH [85], ionic strength [86], temperature [87] or addition of organic co-solvent [88] causes a change in the folding state of the protein chain which is reflected in the distribution of charge states. Typically a native globular protein will populate a narrow distribution of low average charge, whereas its denatured counterpart will feature a broad and highly charged distribution because of the additional sites available for protonation [89] (figure 2). This behaviour can be exploited to monitor the unfolding pathway of proteins [90–92].

Such solution-phase destabilization has been extended to provide a means for studying the composition of protein assemblies [93]. With careful adjustment, solution conditions can be found which effect disassembly of the complex yet stop short of denaturing the constituent protein chains (figure 2). This allows sub-complexes, that is oligomeric species smaller than the original assembly, to be generated in solution and measured in the mass spectrometer. Such equilibrium experiments can therefore be used to reveal the building blocks of assembly [94] and the thermodynamics of the subunit interfaces [95,96]. Furthermore, in the case of heteromeric proteins when multiple sub-complexes can be observed [97], the overlap can be used to generate an interaction map of the protein complex [98,99].

### 2.6. Determining protein quaternary structure and dynamics

The ability of MS to inform on the oligomeric and disassembled states of proteins renders it very attractive for structural biology. The issue of whether such gas-phase measurements can be directly related to the native form has however been a controversial topic [75,100].

#### 2.6.1. Specificity of protein complexes in the gas phase

While it had clearly been demonstrated that specific protein oligomers could be maintained intact within the mass spectrometer, early reports raised the possibility of observing false positives in mass spectra [101,102]. Such non-specific oligomers arise from those electrospray droplets containing more than one analyte molecule, and their artefactual association during droplet fission and evaporation [16]. This effect is concentration-dependent, and therefore the improved sensitivity of modern mass spectrometers as well as the smaller initial droplet sizes resulting from nESI have largely removed the appearance of these unwanted artefacts when determining protein oligomeric state [2]. For cases where experiments necessitate high protein concentrations, methods have been developed to deconvolute the contributions of specific and non-specific protein oligomers [103–105].

Similarly, false positives can be observed in spectra of oligonucleotides [106], and in ligand-binding studies in which the ligand is typically in considerable excess in solution [107,108]. In both cases a large contributor to this effect is the fact that the strength of molecular interactions change upon transfer into the gas phase. Those based on electrostatics, dipoles and polarizability are strengthened owing to the removal of ‘competition’ from water, and conversely hydrophobic associations are weakened [109]. As such the risk of ‘false negatives’, in which contacts present in solution are not represented in the ESI spectra, needs to be considered [110]. Experiments have, however, shown that van der Waal's interactions remaining after dehydration can effectively act to retain contacts driven by water [111]. As such the extent to, and timescale on, which hydrophobic associations can be maintained in the gas phase remains an active area of study [112]. Typically large protein assemblies are held together by a large number of individual contacts, and therefore even those dominated by hydrophobic effects such as membrane protein oligomers [113] or molecular chaperone : target complexes [95] can successfully be interrogated in the gas phase.

#### 2.6.2. Preservation of structure in the gas phase

While protein stoichiometry can be faithfully preserved in the gas phase, the question arises as to whether solution-phase structure is similarly maintained. Various strands of evidence combine to indicate that this is possible, at least on the timescale of typical MS measurements [114]. Protein complexes transmitted through the mass spectrometer and examined ex situ by electron microscopy retain their global topology [115], and in the case of viruses and enzymes can retain infectivity [116] and activity [117], respectively. Similarly, infrared [118] and fluorescence [119] measurements of proteins trapped in vacuum have demonstrated the retention of aspects of solution structure, evidence backed up by molecular dynamics studies [120].

**Figure 4.**
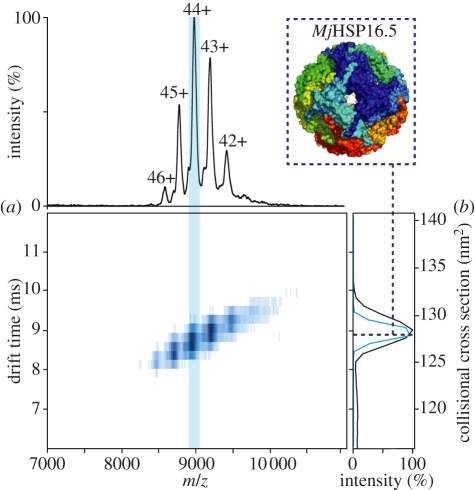
IM–MS spectrum of sHSP *Mj*HSP16.5. (*a*) Two-dimensional plot of drift time versus *m/z* showing the summed spectrum of *Mj*HSP16.5 under native conditions. The MS dimension is projected onto the top panel and shows a charge–state series (42+ to 46+) corresponding to a 397 kDa 24-mer (see the inset). The summation of all the drift times is shown in black (*b*) and the extracted individual drift time distribution for charge state 44+ is shown in blue (*b*). The drift time of an ion can be converted into a rotationally averaged CCS (blue dashed line) which can be compared to the crystal structure (see inset). The CSS of *Mj*HSP16.5 matches that calculated from the crystal structure. The spectrum was acquired as described previously [123].

Perhaps the most compelling evidence comes from ion-mobility (IM) spectrometry measurements, which enable the direct determination of molecular size in terms of a rotationally averaged collisional cross section (CCS), in the gas phase. Evidence suggests the experimental CCS of proteins to be similar to those estimated from atomic constraints [121,122] (figure 4), and that different conformations do not exchange on the timescale of milliseconds [124,125]. Moreover, even the size of fragile protein complexes has been observed to match what is expected from their structure [126]. These observations combine to demonstrate that, on the timescale of typical MS measurements, tertiary and quaternary structures of the protein can be preserved in the gas phase [60].

#### 2.6.3. Structural restraints

The observations outlined above motivate the use of MS for determining structural restraints on protein complexes [4]. Stoichiometry and size information can be obtained from ‘top-down’-type experiments, in which the intact assembly is measured in the gas phase by means of IM–MS. These experiments coupled with the use of gas-phase dissociation can also generate composition and connectivity information. Alternatively, information can be obtained using a ‘bottom-up’ approach, through the interrogation of sub-complexes [127] and folded subunits [128] released from the assemblies in solution under destabilizing conditions. This can provide data on the monomeric, protomeric and oligomeric levels [129], providing valuable information to enable the modelling of protein complex architecture [130]. Furthermore, by combining IM–MS with the additional separation afforded by tandem-MS, candidate structures of polydisperse protein assemblies can be filtered according to their correspndence with measurement [123].

Spatial restraints obtained in this way can be augmented by those obtained from other MS-based approaches [4]. For example, hydrogen/deuterium exchange [131] and oxidative footprinting [132] experiments can be used to reveal secondary structure via solvent accessibility. Limited-proteolysis [133] experiments can provide information as to the domains of the proteins, and cross-linking experiments can reveal protein fold and inter-subunit connectivity [134]. Ultimately, MS-derived structural information can be integrated with restraints obtained from different sources, enabling the modelling of ‘hybrid’ structures which best fit all the available data [135].

#### 2.6.4. Monitoring dynamics

The function of protein complexes hinges not only on their structure, but also on the dynamic processes they undergo, both before and at equilibrium. The speed of analysis and separation afforded by MS renders it well suited to analysing such fluctuations in real time [136,137]. In fact one of the earliest studies to show the preservation of non-covalent interactions in the gas phase monitored in real time the turnover of substrate by the enzyme lysozyme [11]. Furthermore, MS has been used to monitor various other dynamic aspects of proteins, including the folding and conformational fluctuations of protein chains [138].

MS is particularly useful in the study of protein dynamics on the quaternary level, such as monitoring protein complex assembly [139], disassembly [140] and subunit exchange [141]. By virtue of intrinsic mass separation, different oligomeric states can be monitored individually; whereas the quaternary dynamics of individual states can be monitored by incubation with heavier or lighter equivalents (figure 3). An example of this is the incorporation of a ‘mass tag’ introduced by means of mixing homologous proteins [141], or by employing isotopic labelling strategies [142]. Monitoring the disappearance of homo-oligomers and the concomitant formation of hetero-oligomers allows the quaternary dynamics as well as details of their architecture to be ascertained [143].

## 3. Technology and methodology: the state of the art

Since the early experiments in examining non-covalent complexes in the gas phase, instrumentation and methodology have developed rapidly [144]. In this section, we briefly describe the current state of the art of nESI MS analysis of protein assemblies, but also suggest that the reader consult recently published protocols for detailed advice [93,145–149].

### 3.1. Protein preparation

Aqueous solutions of protein complexes are typically prepared at concentrations of 1–10 μM (oligomer), in a volatile buffer. The low concentration guarantees minimal non-specific association during nESI, while the buffer ensures electrochemical effects in the capillary do not affect solution pH. The most commonly used buffered standard is ammonium acetate which, unadjusted, gives a neutral solution even up to high ionic strengths [150], and readily evaporates during ion desolvation. When necessary for the stabilization of the protein assembly, low concentrations of involatile salts or other kosmotropes can be added and still result in tolerable mass spectra [151]. Membrane protein assemblies have specific solubilization requirements, either through stabilization with the minimum amount of a specific detergent [76], or by their release from intact micelles into the gas phase [81].

Spectra of the denatured proteins allow the determination of the masses of the individual subunits, information often essential for establishing oligomeric stoichiometry. These are typically achieved by the addition of organic solvents and acid to the protein solution to degrade the quaternary and tertiary structures. Similarly, identifying the protein chains themselves and the location of post-translational modification through typical proteomic means, either from fragmentation within the mass spectrometer or proteolysis in solution, can provide valuable additional information in the case of purified rather than recombinant sample. Such MS-based proteomics is well established [152], and an important complement to the interrogation of intact protein assembies described here.

### 3.2. Nanoelectrospray ionization

As described in §2.2, MS analyses of protein assemblies are generally performed using nESI owing to the low sample volumes required, and its tolerance of mild interface conditions. nESI is typically performed using borosilicate glass capillaries that have been pulled to form mirco-pipettes. The ends can then be manually clipped under a stereo-microscope to provide an orifice size on the order of 1–5 μm in diameter. Electrospray is initiated by applying a potential difference between the capillary and the inlet to the mass spectrometer, and current is delivered to the solution by either making the capillary conductive via gold coating, or the introduction of a platinum wire. Alternatively nESI can be performed using a chip-based robotic infusion system [153].

### 3.3. Transmission and analysis

As discussed in §2.3, the transmission of large protein assemblies is aided by collisional focusing in the early vacuum stages of the mass spectrometer. Typically this is achieved by reducing the pumping efficiency at the front end of the instrument. Alternative methods exist, and all similarly rely on increasing the number of collisions with background gas experienced by the analyte [32,34]. Additional stabilization of non-covalent complexes can also be achieved in this region by using a curtain gas such as sulphur hexafluoride [154].

These considerations are sufficient for analysing protein complexes on a simple ToF mass spectrometer. The majority of such experiments are performed on Q-ToF instruments, incorporating a modified quadrupole which allows the selection of high *m/z* ions [32,34,50]. Optimum instrument parameters, i.e. operating pressures and voltages, are somewhat sample-dependent, however conditions are typically adjusted to achieve maximum removal of adducts while still maintaining the protein complexes intact.

### 3.4. Ion mobility MS

The current state-of-the-art mass spectrometers for the analysis of protein assemblies incorporate an IM stage, thereby providing two dimensions of separation: effectively mass and size [155]. A number of different means exist to effect IM–MS separation [156] and several have applied to the interrogation of protein multimers, including drift-tube IM [157,158], differential-mobility analysis [159] and energy-loss experiments [160]. The majority of studies on macromolecular assemblies have however employed travelling-wave IM [161], a high-transmission approach which is available on commercial platforms [162,163].

In all cases, the IM measurement can be related to a rotationally averaged CCS of the ion. In the case of travelling-wave experiments this conversion is enabled by calibration using protein standards of known CCS. To this end, a number of protocols [164–166] and CCS databases [167–169] have been published, and it is advisable to use standards of similar mobility to the unknown when performing a calibration [167,170]. It is important to note that even mildly activating conditions within the mass spectrometer, typically used to obtain good quality mass spectra, can cause unwanted structural changes in the protein complexes [57,164]. It is therefore of paramount importance to employ low acceleration voltages prior to IM separation. Additional stabilization can be afforded by charge-reduction [68] or by the addition of kosmotropes [151].

The experimental CCS can be compared with those calculated from atomic structures *in silico*. A number of algorithms exist to achieve this, with the simplest employing a ‘projection approximation’ (PA) [165,171–175]. More sophisticated approaches, including the exact hard-sphere scattering [176] and trajectory methods [177] can also be used. These latter methods, though providing CCS estimates matching experimental values more closely than PA approaches, are more computationally expensive, particularly in the case of the trajectory method [178]. Currently the most convenient strategy for structural biology applications is to employ a scaled PA estimate, as it affords equivalent accuracy and also allows the assessment of coarse-grained molecular models [4].

## 4. The next two decades: MS in structural and dynamical biology

With the dramatic advances since the first measurements of intact non-covalent complexes, and the excellent instrumentation and protocols now available, MS appears to have a large role to play in the evolution of structural and dynamical biology. While anticipating future advances is naturally more difficult than describing past developments, there are several research areas we feel are likely to go beyond just incremental advances to see exciting progress over the coming years.

### 4.1. Standardized and quantitative MS analyses

With the proliferation of structural information stemming from MS experiments, there is an emerging need for the development of experimental standards, and independent criteria for evaluating data quality. Such principles of best practice are either already established or are undergoing development for other structural biology techniques and MS-based proteomics. What form these will take is still unclear, but ultimately we can expect the annotation of protein databases with information from MS-based ‘structural proteomics’ experiments, with the associated requirement for data integrity and deposition.

Equally important to ensuring robust structural information on proteins is the accurate determination of the associated thermodynamic and kinetic parameters that describe their stability and dynamics. As we have described here, MS is well placed to bridge this gap between structural biology and biophysics. Crucial to these efforts is the necessity for MS to accurately reflect the distribution of all molecules in solution [179]. This has been shown to be the case for similar protein species in solution [4]; however, care needs to be taken to overcome the *m/z* dependence of both current mass analysers [180] and detectors [181]. It is probable that future improvements in MS instrumentation will act to overcome these difficulties, ultimately leading to absolute quantification of varied species in solution based on signal intensity alone. This will enable MS to provide not only structural information but also reliably the strength and dynamics of interfaces within diverse macromolecular assemblies.

### 4.2. Structural proteomics through automated multiplexed MS

In order to appropriately characterize the stoichiometry of an unknown protein assembly three crucial elements of information are required: the mass of the intact complex, and the identity and masses of the constituent subunits. As discussed in this article, obtaining the former is now well-established and, building on previous studies [182,183], will probably allow for the automated screening of simple unknowns. The latter two are generally trivial to obtain in the case of recombinantly expressed assemblies, but not in the case of heteromeric complexes isolated from cells, where subunit masses are often considerably different to that expected from genomic databases [184]. It is necessary to perform experiments which separate the protein subunits and in parallel allow their mass measurement and the determination of sequence information [185,186]. We envisage the development in the coming years of MS platforms in which all these levels of information can be obtained in a single multiplexed experiment, thereby providing an automated accurate and reliable means for characterizing protein stoichiometry.

Furthermore, while in this review we have focused on the direct analysis of protein assemblies, there are a plethora of MS-based technologies which can inform on a wide range of structural aspects and timescales [4]. In fact, the vast majority of MS experiments performed on proteins rely on examining the array of peptides produced by enzymolysis of cell extracts or purified components [152,187]. These experiments can be highly automated both in terms of software and hardware [144], and thereby provide a vast amount of data on the sequence level of proteins [188]. In this way, structural probes which have been introduced through, for example, chemical cross-linking, hydrogen/deuterium exchange or oxidative-foot-printing experiments, can be localized. A major goal for MS is to combine these approaches into an integrated structural proteomics platform, enabling the determination of spatial and dynamical restraints spanning the residue to oligomer levels.

### 4.3. Visualizing gas phase ions

In addition to combining these existing MS technologies into a synergistic whole, there are a number of exciting frontiers in the gas-phase visualization of protein assemblies. The opportunities afforded by the possibility of interrogating mass-selected ions, in the absence of solvent background, are significant, and gas-phase spectroscopy of isolated proteins [118,119] and complexes [189] promises to provide considerable insight into their conformation. IM–MS is likely to evolve considerably too, through not only incremental improvements in resolution, but also potentially by dipole alignment in the gas phase [189], or through the use of specific dopants in the IM gas [190].

The use of MS as a high-resolution purification method is likely to prove very useful, allowing ex situ analysis of selectively deposited material by electron microscopy [115]. This will allow the construction of initial models to guide downstream single-particle electron microscopy analysis. Higher resolution structural information on isolated biomolecules is promised by the advent of free-electron laser single-molecule X-ray diffraction [191]. Combining this ability of determining atomic structures with the separation and manipulation afforded by MS represents an exciting frontier for the characterization of heterogeneous macromolecules.

### 4.4. From structural to cell biology

As we have discussed, MS can already contribute significantly towards structural biology, both in isolation and in combination with other techniques. With the continual development of computational structural biology, it is anticipated that ever fewer spatial restraints will be required to produce high-fidelity structures. This is likely to lead to an increasing role for MS, as its generality, speed, and sensitivity will outweigh the fact that it provides fewer restraints than some other structural biology techniques.

Crucial over the coming years are efforts to bridge the gap between structural biology *in vitro*, and the situation *in vivo* [192]. MS has the potential to play an important role in this regard. Already MS-based approaches dominate the field of proteomics (and are likely to play a similar role in metabolomics, lipidomics and glycomics [193]), informing as to the identity, modification and abundance of different proteins in the cell [188]. Furthermore, the high sensitivity of MS allows the interrogation of protein complexes affinity-purified directly from cells [98]. Indeed, when the protein complexes are in high abundance, they can be measured intact directly from diluted crude cell extracts [194], and potentially even from individual cells [195]. The advent of desorption ESI [196], which has been shown to allow the transfer of even protein complexes into the gas phase [197] raises the possibility of probing macromolecular assemblies directly from cell or tissue surfaces. Combining this with the gas-phase separation of different classes of biomolecules in IM-MS spectra [198], leads to the prospect of not only extracting good quality mass spectra of protein complexes despite a high solute background [164], but also the tantalizing prospect of interrogating protein assemblies within the context of their cellular milieu.

## 5. Conclusions

Over the past two decades native MS has evolved to become a structural biology approach of remarkably general utility, providing insights into the composition, architecture, and dynamics of protein complexes. With the realization that the study of the most challenging systems is likely to require a combination of approaches [199,200] and an appreciation of the cellular environment [192], MS will have a crucial role in characterizing the molecular structure, dynamics, and interactions of molecules in the cell.
